# Stratifying prostate cancer patients by relative lymph node involvement: population‐ and modeling‐based study

**DOI:** 10.1002/cam4.776

**Published:** 2016-05-26

**Authors:** Jan T. Poleszczuk, Peter A. Johnstone, Heiko Enderling

**Affiliations:** ^1^ Department of Integrated Mathematical OncologyH. Lee Moffitt Cancer Center & Research InstituteTampaFlorida33612; ^2^ Department of Radiation OncologyH. Lee Moffitt Cancer Center & Research InstituteTampaFlorida33612

**Keywords:** Health services research, lymph node‐positive, mathematical oncology, relative survival

## Abstract

It is estimated that about 10% of new prostate cancer (PCa) cases are lymph node‐positive (LN+). We have previously discussed the role of the inflection point (IP) of an inverse Gompertzian survival curve as a surrogate for disease incurability. In this study, we aimed to stratify curability of different patient cohorts with pathologically positive lymph nodes through modeling survival curves by different percentages of LN involvement (%LN+) postoperatively and calculating associated IPs. From the Surveillance, Epidemiology, and End Results (SEER) database, we selected LN+ PCa patients undergoing radical prostatectomy. Modeling of relative survival curves using inverse Gompertzian kinetics for increasing value of maximal %LN+ involvement allowed stratification of cohort into groups with <10%, 10–40%, and greater or equal to 40% of LN+ out of all LNs sampled. Data were retrieved for 5903 patients. For the entire cohort, relative survival was 96%, 87%, and 76% at 5, 10, and 15 years, respectively. For %LN +, <10% the IP was about 27 years postoperatively. Patients with 10–40% LN+ had an IP at about 10 years; for those with more than 40% LN+, the IP was 7 years. A 10‐year relative survival decreases from 97% for <10% LN+ to 71% for more than 40% LN+. While better therapies for LN + PCa are badly needed, this patient cadre is not homogenous and should be stratified by %LN+ in future clinical trials.

## Introduction

Development of screening techniques has reduced the incidence of prostate cancers (PCa) that have already spread to neighboring lymph nodes (LN+) from about 40% to <10% in the last 50 years 1. However, this still accounts for about 22,000 new cases each year in the US alone 2. The sole recent randomized data for prostate cancer patients with positive lymph nodes after radical prostatectomy (RP) recommends adjuvant hormonal therapy postoperatively 3. However, it has been long understood that degree of LN involvement has a significant role in outcome. Whether microscopic versus gross involvement 4, 5, LN tumor volume 6, presence of extracapsular extension 7 or described by ratio 5, 8, 9, less LN involvement contributes to longer survival than more involvement.

Mathematical modeling provides mechanisms to predict tumor behavior based on cellular signatures, and also to predict patient‐specific treatment options based on retrospective population dynamics. In 2001, we introduced a model for death rates of different cancer patient cadres 10, based on untreated patient populations of breast 11 and cervical cancer 12, and validated model predictions using data of treated patients from the National Cancer Database. We have applied this model to the population of LN+ PCa patients 13. Survival curves from the Surveillance, Epidemiology and End Results (SEER) registry between 1988 and 1993 were modeled using this technique with high accuracy (*R* ^2 ^= 0.998). The model construct is based on a sigmoidal‐shaped inverse Gompertzian model of population death 14, 15. The first part of the curve with a relatively small decrease in survival describes the time frame in which the applied treatment can be considered successful; progression of cancer to advanced stages and the ultimate treatment failure in some cases is reflected in the late exponential decline of the survival curve 13. The concept of the inflection point (IP), that is, the point at which the transition from relatively slow to exponential decline occurs, is central as populations with less severe disease or possessing more effective treatment have a later inflection point 10, 11, 12, 13. Given sufficient follow‐up time, a patient cadre survival curve will exhibit an IP for any treatment that is not 100% successful. Before reaching an IP, potential curative interventions may be applied; after this point, reproducible cure is unlikely 13. It is important to understand that this is true only with respect to existing treatments during the era in question. New therapies of course may provide cure, but that will impact a different cohort of patients.

Our objective was to apply the established inverse Gompertzian modeling framework to analyze the cadre of LN+ PCa patients in greater detail. Based on percentage LN involvement, that is, ratio of positive LNs to all LN sampled, and the concept of the IP, we aim to stratify the LN+ cohort into groups that benefit differently from current treatment techniques.

## Materials and Methods

### Study population

We derived an analytic dataset from the SEER research database (November 2014 submission), consisting of male patients with initial primary prostate cancer (ICD‐O code C61.9) diagnosed in 1988 or later who had undergone radical prostatectomy (surgery specific code 60 and 50 for cases diagnosed before and after 1997, respectively) and with defined numbers of both tested and positive lymph nodes. These data, obtained from a national sample, likely includes hormonal therapy in the disease trajectory. Patient characteristics are summarized in Table 1.

**Table 1:** Whole cohort characteristics

Number of patients (N)	5903
Age	62.48 ± 7.4
Race(%)	
White	83.64
Black	11.54
Other	4.3
Unknown	0.53
Morphology/grade(%)	
Well differentiated; grade I	0.71
Moderately differentiated; grade II	20.94
Poorly differentiated; grade III	76.61
Undifferentiated; grade IV	1.19
Unknown	0.56
Radiation(%)	
No	76.15
Yes	20.63
Unknown	3.22

To ensure that the results of the entire group are indeed generalizable to smaller subcohorts, we created an additional subcohort of 2758 white patients with grade III lesions not receiving radiotherapy. This allowed us to investigate whether other cofactors might confound results of the subsequent analysis. Other patient subcohorts (different races/ethnicities, grades, etc.) had insufficient numbers for analysis.

### Survival analysis

Age‐, race‐, and gender‐matched expected survival for each group was calculated using the Ederer II method 16 using expected survival life tables provided by SEER and distributed with the SEER*Stat software (NCI, Bethesda, MD, USA and Information Management Services Inc., Calverton, MD, USA). Relative survival was estimated by calculating observed survival using Kaplan–Meier estimates and then dividing its value at each time point by the corresponding expected survival 16, 17. Relative survival was adjusted if exceeding 100%. In case of increasing values, correction was made using the value from the previous period.

Standard error for the survival was obtained using the Greenwood formula and statistical significance between each relative survival curves at specific time point was performed using *Z*‐test 17. Survival comparisons between observed survival curves were performed using two‐tailed log‐rank tests.

### Modeling and calculation of inflection point

Relative survival (RS) curves were modeled using inverse Gompertzian kinetics

(1) $$RS=1-b_{1}\exp\left(-b_{2}\exp\left(-b_{3}t\right)\right)$$

with values of parameters *b*_*1*_, *b*_*2*_, and *b*_*3*_ that minimize the sum of squared differences between the model and survival curves at each month of follow‐up (least squares regression). Minimization was performed using genetic and deterministic algorithms implemented in MATLAB (Mathworks Inc., Natic, MA, USA).

In prior publications 10, 13, first differences in the first derivative were used to estimate the derivative of the curves at specific points in time. Here, we calculate this inflection point (IP), that is, the point at each the derivative has a minimal value, using the analytical expression for the derivative

$$\frac{dRS}{dt}=-b_{1}b_{2}b_{3}\exp\left(-b_{2}\exp\left(-b_{3}t\right)\right)\exp\left(-b_{3}t\right)$$

and utilizing the minimum searching procedure implemented in MATLAB, with the initial guess informed by the plot of the derivative.

## Results

### Whole cohort survival and inflection point analysis

**Figure 1:**
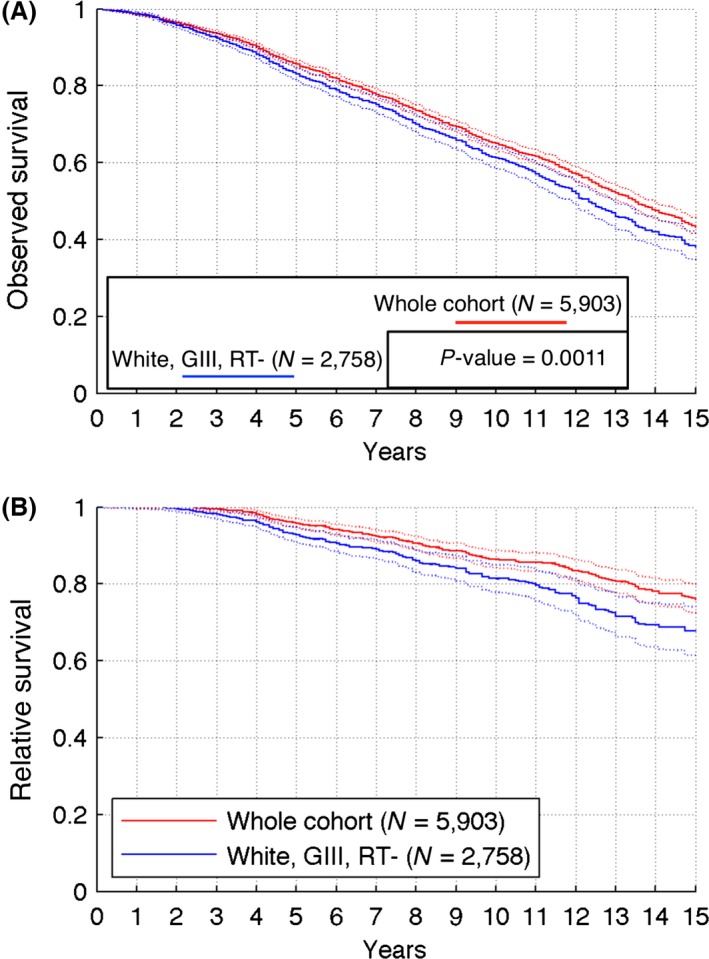
Calculated observed (A) and relative (B) survival curves for the whole analyzed cohort (red solid curve) and subcohort of white patients with grade III tumors who did not receive radiotherapy (blue solid curve). Dotted lines show 95% confidence intervals. *N* = size of the cohort; *P*‐value calculated using two‐tailed log‐rank test.

From the observed survival curve (Fig. 1A), 43.5% of patients will survive 15 years after diagnosis. Relative survival for the entire cohort is 96%, 87%, and 76% at 5, 10, and 15 years, respectively (Fig. 1B).

**Figure 2:**
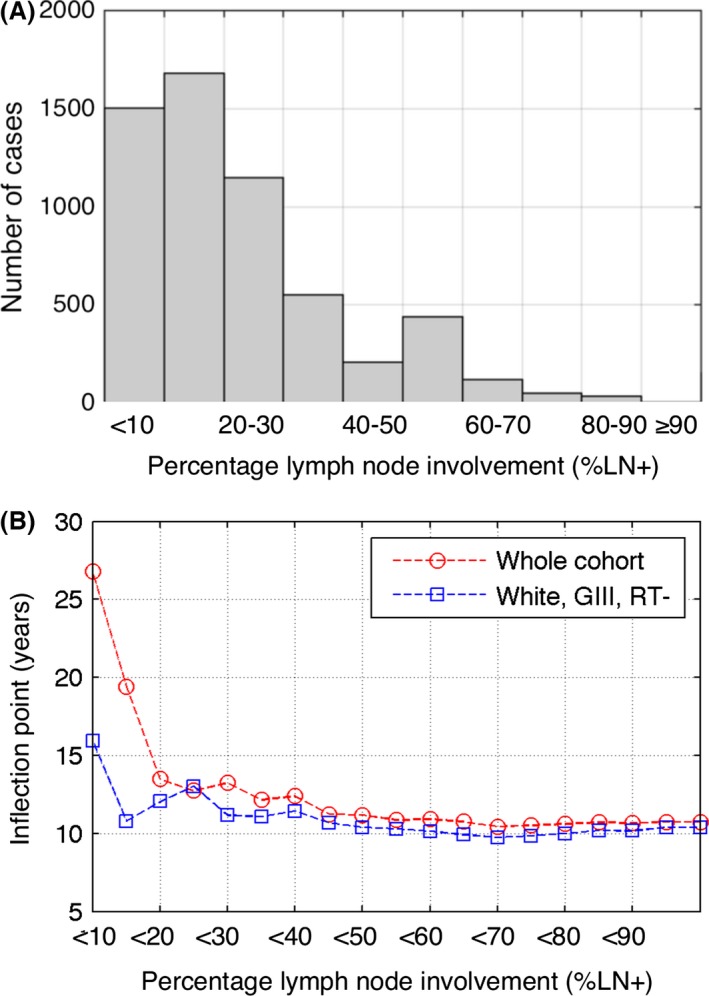
Stratification by the relative number of positive lymph nodes (%LN+). (A) Distribution of %LN+ in the whole cohort. (B) Calculated inflection points resulting from estimated relative survival curves for cohorts generated based on different LN% inclusion criteria.

The left‐skewed distribution of %LN+ in the whole cohort (Fig. 2A) allows creating subsets of patient cohorts for which %LN+ does not exceed a specified cutoff value, which can be as small as 10%. We calculated and modeled relative survival curves for increasing values of %LN+ cutoff. Corresponding inflection points decrease rapidly for low %LN+ cutoff values (Fig. 2B). Extending the cadre of patients having <10% of LN+ to those having <20% of LN+ causes a decrease in IP from initial 26.8 to 13.5 years.

**Figure 3:**
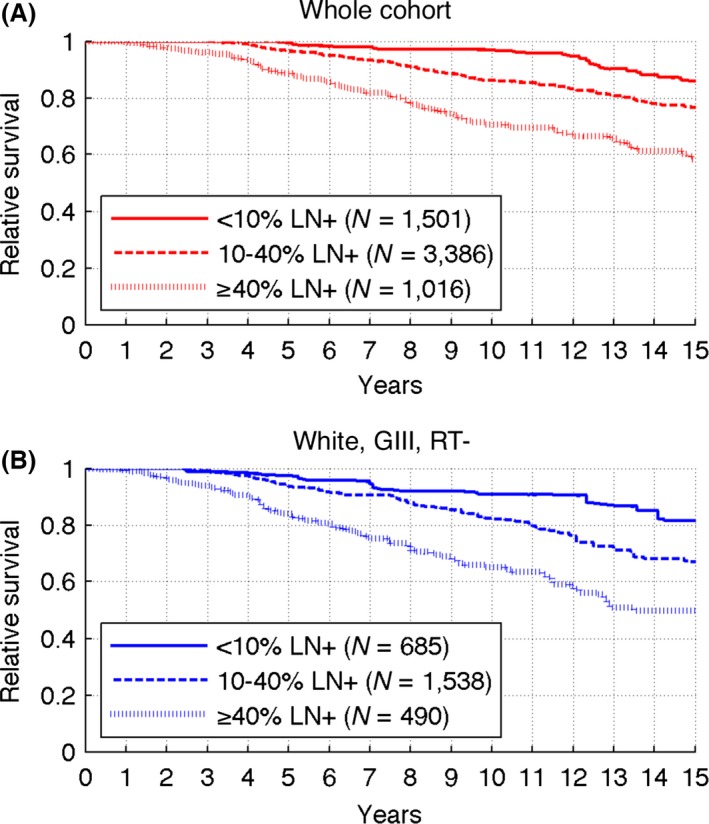
Estimated relative survival curves for %LN stratified cohorts. (A) Whole cohort. (B) White patients with grade III tumors not receiving radiotherapy.

Based on the inflection point analysis presented in Figure 2B, we stratify patients into three groups: (1) %LN + <10%; (2) %LN+ between 10% and 40%; and (3) %LN+ greater or equal than 40%. This grouping resulted in well‐separated RS curves (Fig. 3A), with significantly better prognosis for patients with lower values of %LN+. The estimated relative survival curves for each %LN+ group at 5, 10, and 15 years differ significantly (*P *< 0.05) and the 10‐year relative survival diminishes from 97% for <10% LN+ to 71% for more than 40% LN+ (*P *< 0.001).

**Figure 4:**
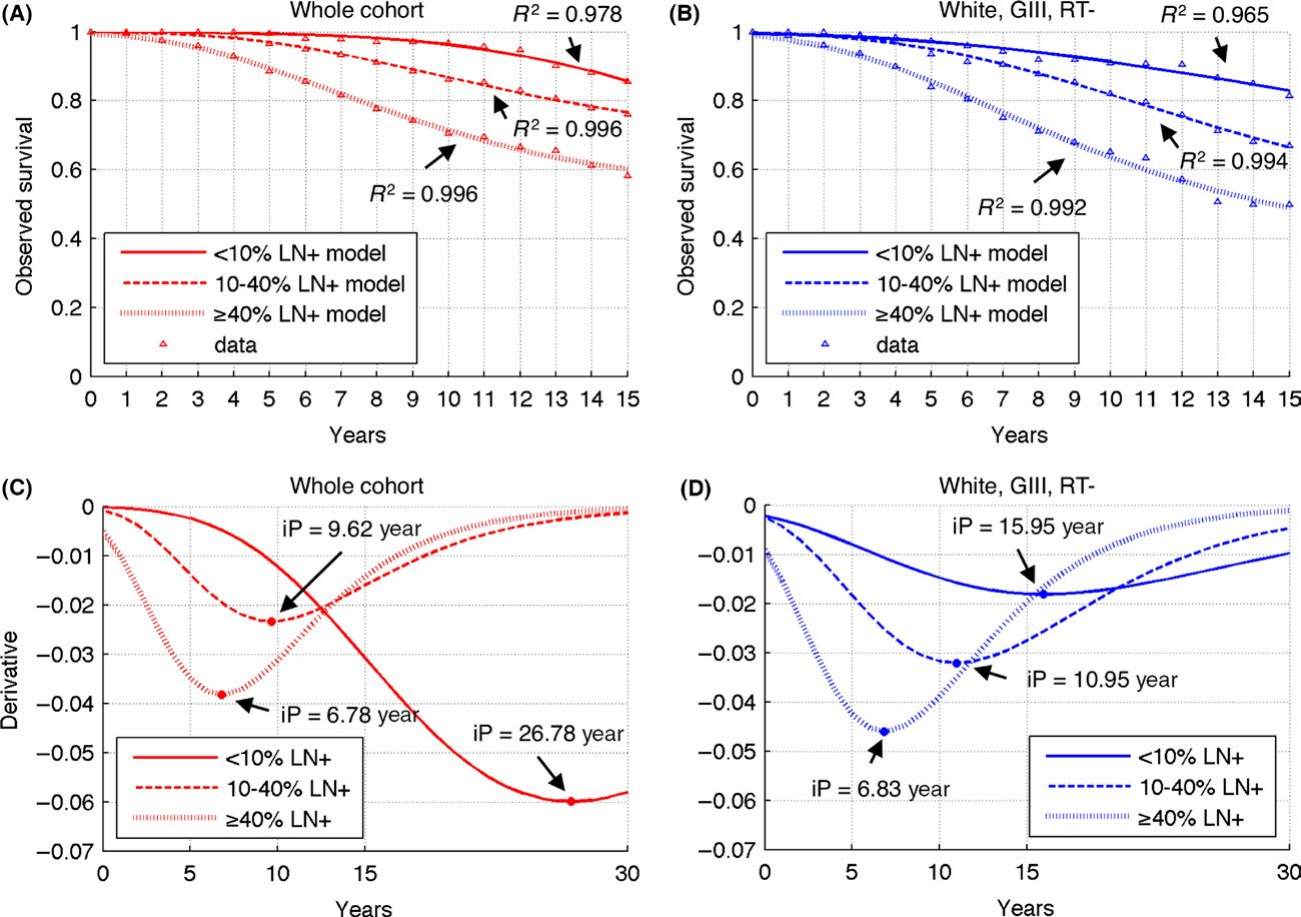
Results of relative survival modeling for each considered %LN+ group. (A), (B) Best fitting inverse Gompertz curves (Eqn. (1)) for parameters summarized in Table 2. (C), (D) Derivatives of fitted curves together with calculated inflection points (circles).

Inverse Gompertzian kinetics (Eq. (1)) provided excellent fit to relative survival curves for each %LN+ group (Fig. 4A, Table 2), with *R* ^*2* ^> 0.97. From the analysis of Eqn. (1), it follows that the inflection points depends only on parameters *b*_*2*_ and *b*_*3*_. Larger *b*_*3*_ values yield smaller IP, while larger *b*_*2*_ yield larger IP; the strongest dependence is on parameter *b*_*2*_. The inflection point varies inversely with increasing %LN+ (Fig. 4C). For %LN+ values <10%, the IP was about 27 years postoperatively for the whole cohort. Patients with 10–40% LN+ had an inflection point at about 10 years; for those with more than 40% LN+, the inflection point was 7 years.

**Table 2:** Estimated parameters for inverse Gompertzian kinetics (Eq. (1)) and all considered patient cohorts

	<10% LN+	10–40% LN+	≥40% LN+
Whole cohort			
*b*_*1*_	1.985	0.337	0.469
*b*_*2*_	9.016	6.15	4.51
*b*_*3*_	0.082	0.189	0.222
White, Grade III, no radiotherapy			
*b*_*1*_	0.513	0.579	0.616
*b*_*2*_	4.64	5.22	4
*b*_*3*_	0.096	0.151	0.203

LN+, positive lymph node.

### Subcohort survival and inflection point analysis

At 5, 10, and 15 years, relative survival for the white, GIII, RT(‐) subcohort was significantly lower than for the whole cohort (*P *< 0.05) with rates of 93%, 81%, and 68%, respectively (Fig. 1B). Distribution of relative LN involvement (%LN+) in the subcohort is qualitatively the same as for the entire cohort, with about 50% less cases in each %LN+ strata presented in Figure 2A. The qualitative behavior of the IP when increasing LN positivity cutoff is also similar as for the entire sample (Fig. 2B). Extending the cadre of patients having <10% of LN+ to those having <20% of LN+ causes a decrease in IP from 15.95 to 10.8 years.

Stratification of white, GIII, RT‐ patients in the same %LN+ groups as the whole cohort also resulted in well‐separated RS curves (Fig. 3B). The differences between relative survival values at 5, 10, and 15 years are also statistically significant, except for the 5‐ and 15‐year points between <10% and 10–40% groups. For that subcohort, the 10 years relative survival decreases from 91% for <10% LN+ to 65% for more than 40% LN+ (*P *< 0.001).

Inverse Gompertzian kinetics (Eq. (1)) again provided excellent fit to relative survival curves for each %LN+ group (Fig. 4B, Table 2) and inflection point varies inversely with increasing %LN+ (Fig. 4D). For %LN+ values <10%, the IP was about 16 years postoperatively. Patients with 10–40% LN+ had an inflection point at about 11 years; for those with more than 40% LN+, the inflection point was 7 years.

## Discussion

Optimal treatment schedules for lymph node‐positive prostate cancers are yet to be defined. Here, we show, using the metric of time to IP, that the population of LN + PCa patients is highly heterogeneous. We developed and analyzed an analytic dataset derived from the SEER research database consisting of male patients with initial primary prostate cancer diagnosed in 1988 or later undergoing RP. These cohort data take into account the use of adjuvant therapies in the LN+ population in the US between 1988 and 2012. Hormonal therapy may safely be assumed to have been used diffusely through this population. Newer adjuvant therapies, on the other hand may not be expected to impact these survival data.

We stratified this cadre of patients using the percentage LN involvement (the ratio of LN+ to all LNs examined). The proposed grouping resulted in well‐separated relative survival curves, with significantly better prognosis for the patients with lower values of %LN+. In the general population of LN+ patients, the 10‐year relative survival diminishes from 97% for <10% LN+ to 71% for more than 40% LN+. This is reflected also in a drop in the estimated time to IP: from about 27 years for <10% LN+ to about 7 years for more than 40% LN+. Importantly, when stratifying inflection points by number of positive LN, there is a light predictive capability of 1, 2, 3, or ≥4 positive LNs (see Fig. S1). This is less useful than percentage LN involvement (%LN+).

Analysis of the subcohort of grade III, white patients who did not receive radiotherapy shows the same qualitative behavior, that is, the larger the LN involvement, the worst is the prognosis. Moreover, for 10–40% LN+ and more than 40% LN+, the estimated IP values in the subcohort are the same as in the whole cohort. However, there is a large (almost 11 years) difference in the IP for patients with less than 10% LN+, which could indicate that race, RT, and/or high‐grade tumors (Grade III) may influence the inflection point when lymph node involvement is low.

Conceptually, the inflection point (IP) represents uniquely a calculated endpoint independent from biochemical control or survival. The IP, based on the Gompertz model, represents the point at which cancer with different degree LN+ become incurable. This then allows better informed decisions about potentially curative therapies. The population of men with LN+ post‐op could be stratified into IP‐dependent categories with appropriate therapeutic approaches.

In summary, our study suggests that clinical protocols for LN+ PCa should consider patient heterogeneity, and %LN+ in particular. Future clinical trials for LN+ patients should stratify patients by %LN+ to remove survival biases and allow identification of potentially curative therapies for LN+ PCa.

## Conflict of Interests

None declared.

## Supporting information


**Figure S1.** (A) Estimated relative survival curves for LN+ stratified cohorts. (B) Derivatives of fitted curves together with calculated inflection points (circles).

## References

[1] Swanson, G. P. , I. M. Thompson , and J. Basler . 2006 Current status of lymph node‐positive prostate cancer: incidence and predictors of outcome. Cancer 107:439–450. 16795064 10.1002/cncr.22034

[2] Siegel, R. L. , K. D. Miller , and A. Jemal . 2015 Cancer statistics, 2015. CA Cancer J. Clin. 65:5–29. 25559415 10.3322/caac.21254

[3] Messing, E. M. , J. Manola , M. Sarosdy , G. Wilding , E. D. Crawford , and D. Trump . 1999 Immediate hormonal therapy compared with observation after radical prostatectomy and pelvic lymphadenectomy in men with node‐positive prostate cancer. N. Engl. J. Med. 341:1781–1788. 10588962 10.1056/NEJM199912093412401

[4] Smith, J. A. , and R. G. Middleton . 1985 Implications of volume of nodal metastasis in patients with adenocarcinoma of the prostate. J. Urol. 133:617–619. 3920406 10.1016/s0022-5347(17)49112-9

[5] Steinberg, G. D. , J. I. Epstein , S. Piantadosi , and P. C. Walsh . 1990 Management of stage D1 adenocarcinoma of the prostate: the Johns Hopkins experience 1974 to 1987. J. Urol. 144:1425–1432. 1700157 10.1016/s0022-5347(17)39759-8

[6] Cheng, L. , E. J. Bergstralh , J. C. Cheville , J. Slezak , F. A. Corica , H. Zincke , et al. 1998 Cancer volume of lymph node metastasis predicts progression in prostate cancer. Am. J. Surg. Pathol. 22:1491–1500. 9850175 10.1097/00000478-199812000-00006

[7] Griebling, T. L. , D. Ozkutlu , W. A. See , and M. B. Cohen . 1997 Prognostic implications of extracapsular extension of lymph node metastases in prostate cancer. Mod. Pathol. 10:804–809. 9267823

[8] Bader, P. , F. C. Burkhard , R. Markwalder , and U. E. Studer . 2003 Disease progression and survival of patients with positive lymph nodes after radical prostatectomy. Is there a chance of cure? J. Urol. 169:849–854. 12576797 10.1097/01.ju.0000049032.38743.c7

[9] Schmidt, J. D. , R. P. Gibbons , A. Bartolucci , and G. P. Murphy . 1989 Prognosis in stage D‐1 prostate cancer relative to anatomic sites of nodal metastases. National Prostatic Cancer Treatment Group. Cancer 64:1743–1746. 2790688 10.1002/1097-0142(19891015)64:8<1743::aid-cncr2820640831>3.0.co;2-u

[10] Riffenburgh, R. H. , and P. A. S. Johnstone . 2001 Survival patterns of cancer patients. Cancer 91:2469–2475. 11413539

[11] Johnstone, P. A. , M. S. Norton , and R. H. Riffenburgh . 2000 Survival of patients with untreated breast cancer. J. Surg. Oncol. 73:273–277. 10797344 10.1002/(sici)1096-9098(200004)73:4<273::aid-jso15>3.0.co;2-h

[12] Adriano, E. , J. M. Jagoe , T. Harrison , R. H. Riffenburgh , and P. A. Johnstone . 2003 Survival of patients with untreated cervical carcinoma. Am. J. Clin. Oncol. 26:369–373. 12902888 10.1097/01.COC.0000026909.73608.88

[13] Johnstone, P. A. , R. H. Riffenburgh , P. J. Rossi , V. Assikis , V. A. Master , and A. B. Jani . 2007 Applying population dynamics modeling to patients with lymph node positive prostate cancer. J. Urol. 178:1952–1955. 17868720 10.1016/j.juro.2007.07.017

[14] Gompertz, B. 1825 On the nature of the function expressive of the law of human mortality, and on a new method of determining the value of life contingencies. Philos. Trans. R. Soc. Lond. B Biol. Sci. 115:513–585. 10.1098/rstb.2014.0379 PMC4360127 25750242

[15] Winsor, C. P. 1931 The Gompertz curve as a growth curve. Proc. Natl Acad. Sci. USA 18:1–8. 16577417 10.1073/pnas.18.1.1 PMC1076153

[16] Hakulinen, T. , and H. H. Abeywickrama . 1985 A computer program package for relative survival analysis. Comput. Programs Biomed. 18:197–207. 3839736 10.1016/0010-468x(85)90011-x

[17] Parkin, D. M. , and T. Hakulinen . 1991 Cancer registration: principles and methods. Analysis of survival. IARC Sci. Publ. 95:159–176. 1894319

