# Supplementary material for: Stratifying prostate cancer patients by relative lymph node involvement: population‐ and modeling‐based study
Source: Cancer Med. 2016 May 26;5(8):1850–5. doi: 10.1002/cam4.776 (PMC4884636; doi:10.1002/cam4.776)
Supplement: Supplementary file 1 — Figure S1. (A) Estimated relative survival curves for LN+ stratified cohorts. (B) Derivatives of fitted curves together with calculated inflection points (circles). [file CAM4-5-1850-s001.docx]

Supplementary Materials

**Stratifying prostate cancer patients by relative lymph node involvement: population- and modeling-based study**

*1. Stratification by number of positive lymph nodes*

In this section we show the results of the very same analysis as performed in the main text, but instead of stratifying patients on the basis of percentage LN involvement we used absolute number of positive lymph nodes (LN+). To this extent we divided the whole cohort into 4 distinct groups: 1 LN+, 2 LN+, 3 LN+ and ≥ 4 LN+.

This grouping resulted in not as well separated relative survival (RS) curves as in the case of stratification by %LN+ (compare Fig. S1(a) and Fig. 4). The differences in the RS between 1, 2, and 3 LN+ groups were not significantly different (p-val>0.1; Z-test at 5, 10 and 15 years), except for 10 and 15 years points between 1 and 2 LN+ groups (p-val<0.05). The prognosis for ≥ 4 LN+ is, however, significantly worse than for other LN+ groups (p-val<0.01 in all cases).

Poor separation in the relative survival curves resulted in small differences in the estimated inflection points (IPs) between considered LN+ groups (Fig. S1(b)). Hence, from additional analysis it follows that stratification by LN+ is less useful than by percentage LN involvement (%LN+).


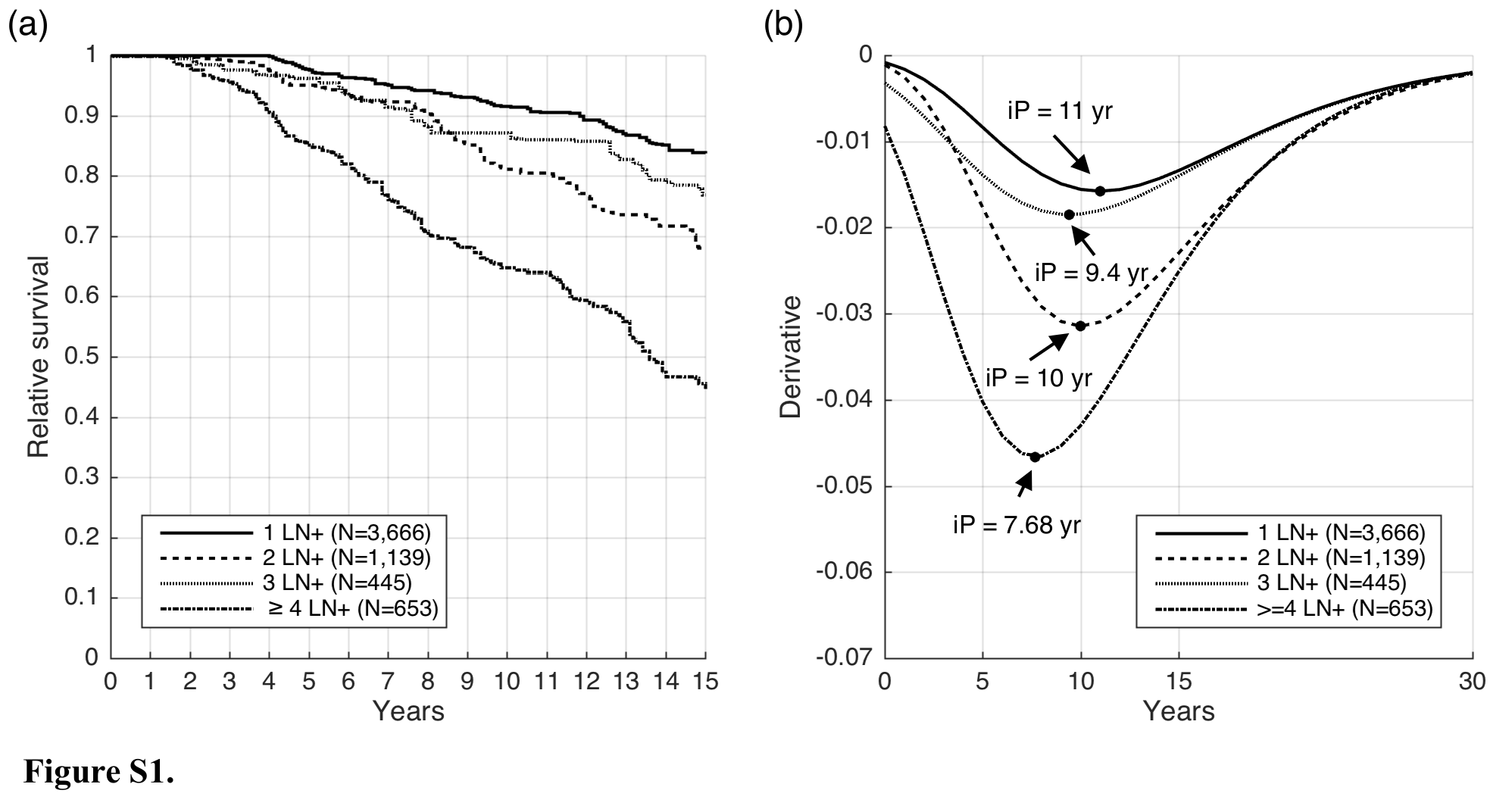
(a) Estimated relative survival curves for LN+ stratified cohorts. (b) Derivatives of fitted curves together with calculated inflection points (circles).
